# Critical care medicine in the French Territories in the Americas: Current situation and prospects

**DOI:** 10.26633/RPSP.2021.46

**Published:** 2021-04-28

**Authors:** Hatem Kallel, Dabor Resiere, Stéphanie Houcke, Didier Hommel, Jean Marc Pujo, Frederic Martino, Michel Carles, Hossein Mehdaoui, Antilles-Guyane Association of Critical Care Medicine

**Affiliations:** 1 Cayenne General Hospital Cayenne French Guiana Cayenne General Hospital, Cayenne, French Guiana; 2 Martinique University Hospital Fort-de-France Martinique Martinique University Hospital, Fort-de-France, Martinique; 3 Guadeloupe University Hospital Pointe-à-Pitre Guadeloupe Guadeloupe University Hospital, Pointe-à-Pitre, Guadeloupe

**Keywords:** Critical care, tropical medicine, French Guiana, Guadeloupe, Martinique, Cuidados críticos, medicina tropical, Guyana Francesa, Guadalupe, Martinica, Cuidados críticos, medicina tropical, Guiana Francesa, Guadalupe, Martinica

## Abstract

Hospitals in the French Territories in the Americas (FTA) work according to international and French standards. This paper aims to describe different aspects of critical care in the FTA. For this, we reviewed official information about population size and intensive care unit (ICU) bed capacity in the FTA and literature on FTA ICU specificities. Persons living in or visiting the FTA are exposed to specific risks, mainly severe road traffic injuries, envenoming, stab or ballistic wounds, and emergent tropical infectious diseases. These diseases may require specific knowledge and critical care management. However, there are not enough ICU beds in the FTA. Indeed, there are 7.2 ICU beds/100 000 population in Guadeloupe, 7.2 in Martinique, and 4.5 in French Guiana. In addition, seriously ill patients in remote areas regularly have to be transferred, most often by helicopter, resulting in a delay in admission to intensive care. The COVID-19 crisis has shown that the health care system in the FTA is unready to face such an epidemic and that intensive care bed capacity must be increased. In conclusion, the critical care sector in the FTA requires upgrading of infrastructure, human resources, and equipment as well as enhancement of multidisciplinary care. Also needed are promotion of training, research, and regional and international medical and scientific cooperation.

## RESUMEN

Los hospitales en los territorios franceses de la Región de las Américas funcionan según las normas francesas e internacionales. El objetivo de este artículo es describir distintos aspectos de los cuidados intensivos en los territorios franceses. Para ello, hemos revisado los datos oficiales sobre el tamaño de la población y el número de camas de las unidades de cuidados intensivos (UCI), así como la bibliografía sobre algunos aspectos específicos de las UCI, en los territorios franceses. Las personas que viven en los territorios franceses, o que están de visita en ellos, están expuestas a riesgos específicos: principalmente traumatismos graves causados por el tránsito, envenenamiento por mordeduras, heridas de bala o por apuñalamiento, y enfermedades infecciosas tropicales emergentes. La atención de estos traumatismos y enfermedades puede requerir conocimientos específicos y cuidados intensivos. Sin embargo, no hay suficientes camas de UCI en los territorios franceses. De hecho, hay 7,2 camas de UCI por 100 000 habitantes en Guadalupe, 7,2 en Martinica y 4,5 en Guayana Francesa. Además, los pacientes gravemente enfermos que viven en zonas remotas a menudo tienen que ser trasladados, normalmente por helicóptero, lo que retrasa su ingreso en la unidad de cuidados intensivos. La crisis de la COVID-19 ha puesto de manifiesto que el sistema de atención de salud en los territorios franceses no está preparado para enfrentarse a una epidemia de estas dimensiones y que debe aumentarse la capacidad hospitalaria de las unidades de cuidados intensivos. En conclusión, el sector de los cuidados intensivos en los territorios franceses tiene que mejorar su infraestructura, recursos humanos y equipamiento, así como perfeccionar la atención multidisciplinaria. También es necesario promover la capacitación, la investigación y la cooperación médica y científica, tanto regional como internacional.

## RESUMO

Os hospitais nos territórios ultramarinos franceses nas Américas funcionam segundo os padrões franceses e internacionais. O objetivo deste artigo é descrever os diversos aspectos da atenção intensiva nesta região. Analisamos os dados oficiais relativos ao tamanho da população e ao número de leitos de unidade de terapia intensiva (UTI) nestes territórios junto com uma revisão da literatura científica sobre as características particulares destes centros de terapia intensiva. Os residentes locais ou visitantes dos territórios ultramarinos franceses nas Américas são expostos a riscos específicos, sobretudo acidentes de trânsito graves, envenenamentos por animais peçonhentos, ferimentos por armas brancas ou armas de fogo e doenças infecciosas tropicais emergentes que requerem conhecimento especializado e atenção intensiva. Porém, não há leitos suficientes de UTI nos territórios ultramarinos franceses nas Américas: são 7,2 leitos de UTI por 100.000 habitantes em Guadalupe, 7,2 na Martinica e 4,5 na Guiana Francesa. Ademais, em áreas remotas, os pacientes em estado crítico frequentemente precisam ser transferidos por helicóptero, o que causa demora na internação em UTI. A crise da COVID-19 demonstra o despreparo do sistema de saúde para enfrentar a pandemia e a necessidade de aumentar o número de leitos de UTI nestes territórios. Em conclusão, é imprescindível modernizar a infraestrutura e os equipamentos, capacitar melhor os recursos humanos e melhorar a atenção multidisciplinar. Incentivar a formação profissional, pesquisa e cooperação médico-científica regional e mundial é também fundamental.

**FIGURE 1.**
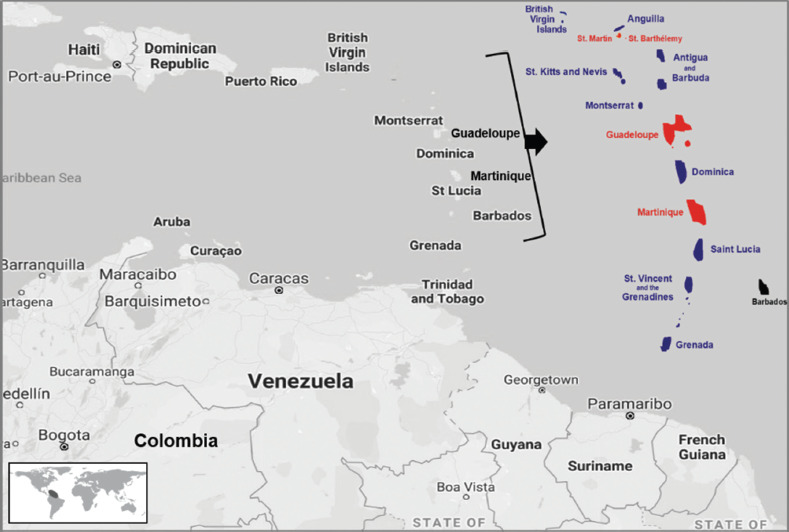
Map of French Territories in the Americas

The French Territories in the Americas (FTA)—French Guiana, Guadeloupe, and Martinique—are all located in the intertropical zone between latitudes 4° and 16° north. They comprise two island regions, Martinique and Guadeloupe (and their dependencies Saint Martin, Saint Barthélemy, and the Guadeloupe archipelago), and a continental region of the Amazon rainforest area of South America, French Guiana (Figure 1).

In France, intensive care units (ICUs) are designed to take care of patients presenting or likely to present with organ failure and requiring artificial organ support, such as mechanical ventilation, catecholamines, and dialysis (circulaire DHOS/SDO n° 2003-413 du 27 août 2003 article R. 712-90). Since there is no common definition of an ICU between countries, schematically, ICUs in France are defined as units where patients can receive mechanical ventilation.

Hospitals in the FTA work according to international, European, and French standards. They are an illustration of a paradox whereby hospitals in a high-income country are serving a significant part of the population with low income and in a situation of precariousness. Regarding ICUs, there are not enough available data covering activity indicators. The interval between the onset of first symptoms and admission to the ICU can be long and therefore may constitute in itself a risk for patients. Many factors can be involved, but the main reasons are the delay of medical transportation from areas often accessible only by air (plane or helicopter) or by boat, the multidisciplinary care time in the emergency sector, which does not necessarily have all the resources immediately accessible, and the lack of ICU beds.

The aim of this paper is to describe different aspects of critical care in the FTA. It focuses on the impact of social and environmental determinants on health and the need for more intensive care beds. It also discusses the prospects for upgrading infrastructure and human resources and on promoting research.

## POPULATION CHARACTERISTICS

The estimated population size in 2019 was 400 000 inhabitants in Guadeloupe (including Saint Martin island), 358 749 in Martinique, and 290 691 in French Guiana (www.insee.fr). In terms of population growth, French Guiana is the most dynamic region, with an average yearly growth of 2.3%, whereas the migratory balance sheet is negative in Martinique and Guadeloupe. This is partly because French Guiana has a younger population: around 50% are under 25 years compared with less than 30% in Guadeloupe and Martinique. Further, Martinique and Guadeloupe benefit from a significant influx of tourists, whereas French Guiana faces immigration, sometimes clandestine, from its neighboring countries (Brazil and Suriname), from the Caribbean arc, mainly migrants from Haiti, and more recently from the Middle East (Palestine, Syria, and others). Population flows, whether travelers or migrants, play a significant role in the development of epidemics and/or emerging pathologies (1). Furthermore, French Guiana is the French overseas territory that registers the most asylum requests (40%), the vast majority of which are refused, leading to numerous undocumented foreign residents, most often without solidarity medical insurance such as the State Medical Aid (AME in French) or the Complementary Universal Medical Coverage (CMUC in French). Thus, they contribute significantly to precariousness and the high poverty rate of the population. Indeed, in 2015, the National Institute of Statistics and Economic Studies (www.insee.fr) estimated the poverty rates at 19% in Guadeloupe, 21% in Martinique, and 44% in French Guiana. Obviously, precariousness and poverty are leading causes of renouncement of health care and contribute to longer length of hospital stay and hospital occupancy rate (2). It is also important to emphasize the impact of cultural diversity and public confidence and practices on the spread of illness.

## HOSPITAL NETWORK

In the FTA, the public hospital network encompasses several sites across four main locations in Martinique, six in Guadeloupe, and three in French Guiana, with one referral hospital in each geographic department. Even though Martinique and Guadeloupe hospitals are university health facilities, Cayenne hospital is still a general hospital, despite seven units being led by university professors (intensive care, infectious diseases, dermatology, public health, laboratory, pediatrics, and neurology). All three hospitals receive residents and medical trainees within their medical course and participate, at the same level, in the university education.

**FIGURE 2.**
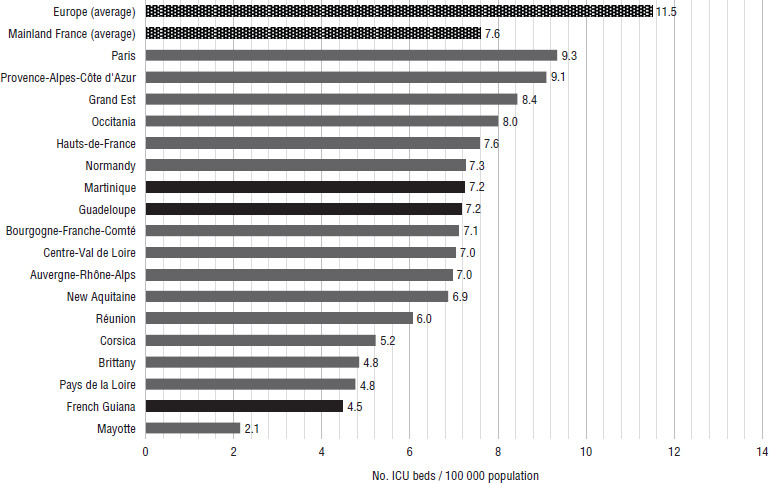
Number of intensive care beds per 100 000 population by departments of France

***Source***: Prepared by the authors using data on the number of intensive care beds from the annual health establishments statistics (SAE) database published in 2018 by the French Ministry of Health; population size from the database published in 2020 by the National Institute of Statistics and Economic Studies (INSEE); and data on Europe from Rhodes et al. (4).

First response for acute medical care is through regional call centers for emergencies: SAMU 971 in Guadeloupe, SAMU 972 in Martinique, and SAMU 973 in French Guiana. Although the travel time to the nearest facility with ICU beds can be about 10 to 20 minutes by car in the urban areas, it can be up to 100 minutes by helicopter from isolated areas. These delays are much higher than those reported in mainland France (17 minutes) (3).

## CRITICAL CARE BED CAPACITY

There is also an ICU in each geographic department, including in those hospitals. In 2020, we identified a total of 66 beds dedicated to intensive care in all three territories, distributed as follows: 7.2 ICU beds/100 000 population in Guadeloupe, 7.2 in Martinique, and 4.5 in French Guiana. Figure 2 shows the inequality of distribution of intensive care beds by the departments of France. These differences are too large to be explained purely by differences in the characteristics of the populations and can contribute to high case fatality rates (3). In 2020, the National Professional Council on Intensive Care Medicine (CNP MIR) judged that the rate of intensive care beds in France was very low and must be upgraded to 10 ICU beds/100 000 population, representing at least 3% of hospitalization capacity (5). Critical care capacity in the overseas French departments is not cited in that report.

The ICU beds in the three departments ensure the management of 2 262 patients annually on average. The ICU characteristics are summarized in Table 1.

Seriously ill patients admitted to local hospitals or remote health facilities will, therefore, have to then be transferred to the ICU, which is only possible by air transportation, most often by helicopter. Moreover, some medical specialties (neurologic or cardiologic surgery, pediatric ICU, severe burns) or technologies (circulatory assistance, extracorporeal membrane oxygenation [ECMO]) are only available in one or two referral sites or are missing from the entire region (e.g., interventional radiology). It is usual to medevac, for a second time, patients to the most appropriate health facility, which can either be among the three referral hospitals in the FTA or in mainland France. The use of air transport (regular or special flights; airplane or helicopter) is common. Medevacs are ensured by SAMU 971, 972, and 973. In the last five years, SAMU 971 ensured 180 medevacs annually on average, SAMU 972 ensured 115, and SAMU 973 ensured 242. The flight to mainland France is approximately 9 hours from French Guiana and 8 hours from Martinique or Guadeloupe; 3.5 hours from French Guiana to Martinique or Guadeloupe; and 0.5 hours from Martinique to Guadeloupe and vice versa.

**TABLE 1.** Intensive care unit characteristics based on data collected in the last five years

	French Guiana	Martinique	Guadeloupe
No. hospital beds	700	1 600	782
No. ICU beds	13	26	27
Ratio ICU/total hospital beds (%)	1.8	1.6	3.4
No. hospitalizations in ICU/year	362	900	1 000
Occupation rate in ICU	83.6	85	90
SAPS II	44	NA	44
Medical type (%)	67.3	50	60
Surgical type (%)	32.7	50	10
Trauma type (%)	22.5	10	30
Mechanical ventilation (%)	60.0	80	70
Dialysis (%)	19.2	25	40
Catecholamines (%)	53.7	30	60
Mean LOS (days)	11.2	9	7
Mortality rate (%)	23.9	20	29

***Notes:*** ICU, intensive care unit; SAPS II, Simplified Acute Physiology Score (6); LOS, length of stay; NA, not available.

***Source:*** Table prepared by authors based on data from the departments of medical information of the French Territories in the Americas.

In addition to patient care, hospitals face logistic issues, the management of which is much more challenging than in mainland France. Indeed, getting medical devices or consumables that conform to European standards from neighboring countries might not be an option, which instead entails ordering them from mainland France with long delivery times as a consequence (on average six to eight weeks).

## CRITICAL CARE BED CAPACITY DURING THE COVID-19 CRISIS AND BEYOND

The COVID-19 crisis has shown that the critical care capacity in the FTA is unready to face such an epidemic. In recent years there has been a growing awareness of the increasing incidence of emergent tropical disease outbreaks. It was hoped that lessons learned during the dengue, chikungunya, and Zika epidemics would serve as an impetus for a revised approach in the strategic planning for critical care readiness in the FTA.

Faced with the COVID-19 epidemic, hospitals in the FTA have significantly expanded their capacities and ensured availability in staff and equipment. As a result, French Guiana has added 35 acute care beds (+270%), Martinique 31 (+119%), and Guadeloupe 11 (+41%). This bed capacity expansion has been organized thanks to the local workforces, the French Ministry of Health and local authorities, regional cooperation, and volunteer nurses and doctors.

After the COVID-19 crisis, a gradual increase in intensive care bed capacity is planned in accordance with the recommendations of the French authorities and the French Society of Intensive Care (5). The target capacity is about 10 ICU beds/100 000 population and 3% of total hospital beds.

## SPECIFICITIES IN CRITICAL CARE

Specificities in critical care comprise the range of infectious diseases, toxicology, envenoming, in addition to a significant rate of cardiovascular diseases and stroke events. Indeed, the diversity of fauna, flora, and ecosystems, as well as the tropical climate with cyclonic components (Guadeloupe and Martinique) and very high rainfall (French Guiana), result in exposure to certain tropical infectious diseases (Table 2) (7, 8), which may require specific knowledge and critical care management. In addition, the high level of forestry and aquatic activities, whether professional, military, or recreational, can result in traumatic accidents with specific injuries. Severe stab or gunshot wounds are frequent and reflect a high level of delinquency and crime in the FTA. Indeed, during the last five years, 450 gunshot injuries were recorded only in Cayenne, French Guiana. Envenoming, mainly snake bites, is frequent in Martinique and French Guiana (9–11), and its treatment requires specific knowledge and a critical care environment (11, 12). Moreover, the prevalence of obesity, diabetes, and cardiovascular diseases in the FTA is among the highest in France (13–15). These are responsible for neurological, renal, and cardiac complications and are common reasons for hospitalization in ICU.

In addition to these specific diseases, the FTA have a high frequency of traffic injuries, accounting for more than 30% of admissions in the three ICUs.

**TABLE 2.** Tropical infectious diseases in the French Territories in the Americas

Tropical disease	Microorganism	Epidemiology
**All French Territories in the Americas**		
Leptospirosis	Leptospira icterohaemorrhagiae	26 cases/10^5^ inhabitants
Zika virus disease	Zika virus	Endemic with epidemic peaks
Chikungunya	Chikungunya virus	Endemic with epidemic peaks
**Martinique and Guadeloupe**		
Melioidosis	Burkholderia pseudomallei	12 cases reported
**French Guiana**				
Q fever	Coxiella burnetii	37–150 cases/10^5^ inhabitants
Histoplasmosis	Histoplasma capsulatum	Sporadic
Amazonian toxoplasmosis	Toxoplasma gondii	Sporadic (since 2002)
Hantavirus pulmonary syndrome	Maripa hantavirus	6 cases reported since 2008
Yellow fever	Yellow fever virus	Endemic
Malaria	*Plasmodium falciparum* (95%), *P. vivax*	Endemic (<500 cases/year)
Cryptococcosis	Cryptococcus sp, C. gattii	Leading cause of encephalitis in French Guiana
Venezuelan equine encephalitis	Tonate virus	Rare
Rabies	Lyssavirus	Rare
Chagas disease	Trypanosoma cruzi	Endemic

***Source***: Table prepared by the authors based on data from Kallel et al. (7) and Melot et al. (8).

## TRAINING

The French West Indies University’s Faculté de Médecine Hyacinthe Bastaraud is based in Pointe-à-Pitre, Guadeloupe, with two other remote sites in Martinique and French Guiana. Professors working in the three sites meet regularly, as well as with students through classroom presentations and Internet videoconferencing. Each year, the French West Indies University graduates 150 doctors of medicine, 10 specialist doctors in emergency medicine, and 10 in anesthesiology.

There are three schools in the FTA that train nurses and anesthetist nurses. During their studies, student internships are ensured in the hospitals. Each year, the three schools graduate 306 nurses and 15 anesthetist nurses.

## INFECTION CONTROL AND BACTERIAL RESISTANCE

In the FTA, hygiene, bacterial resistance, and antibiotic consumption are monitored, studied, and managed according to national programs and standards. According to the national surveillance network, high levels of antibiotic resistance and antibiotic consumption are recorded in the FTA (https://invs.santepubliquefrance.fr). Indeed, in this region, the incidence rate of extended-spectrum β-lactamase producing Enterobacteriaceae (ESBL-PE) is among the highest in the world, at up to 51% for *Klebsiella pneumoniae* and up to 18% for *Escherichia coli* (16, 17). Conversely, a low level of methicillin-resistant *Staphylococcus aureus* is recorded (18). The regional surveillance network in ICUs is planned to take appropriate measures to prevent the spread of drug-resistant bacteria.

## RESEARCH

The FTA face infectious diseases, indigenous forms of opportunistic or tropical diseases, some chronic diseases with a genetic predisposition (sickle cell disease), environmental disaster (invasion of *Sargassum* seaweed), and the health consequences of specific environmental pollution (chlordecone in the West Indies, mercury in French Guiana) (19–22). Monitoring and research needs are therefore important and are attractive for researchers and students. To promote this, local research structures, regional cooperation, national programs, and international medical and scientific cooperation are already established. Several Caribbean countries are currently working together on different projects. However, to progress significantly on knowledge of tropical epidemiology, an adequate flow of researchers and partnerships should be strengthened with universities of neighboring countries, national institutions such as the National Institute of Health and Medical Research (INSERM), the Institut Pasteur (located in Guadeloupe and French Guiana), the Research and Development Institute (IRD) (located in French Guiana and Martinique), University Hospital Centers in metropolitan France (e.g., Assistance Publique des Hôpitaux de Paris), and the National Institute for Public Health Surveillance.

## COOPERATION WITH NEIGHBORING COUNTRIES

**Martinique and Guadeloupe.** The accession of Martinique and Guadeloupe to the Organization of Eastern Caribbean States as associate members has now drawn the English-speaking political directorate into the existing informal cooperation mechanism. The promotion of regional medical cooperation has consequently seen the coming together of agencies such as the Pan American Health Organization (PAHO), the French Regional Health Agency (ARS), the European Union’s INTERREG funding mechanism, the University Hospital of Martinique (CHUM), the Territorial Collectivity of Martinique, the Territorial Collectivity of Saint Martin, and the Department of Guadeloupe to facilitate training and teaching in intensive care.

On an as-needed basis, specific training in areas such as Ebola and Zika virus disease management has been undertaken for medical staff across the wider Caribbean, with sessions conducted in both Martinique and Saint Lucia with the participation of 42 nurses and 5 medical doctors. The main objective of the training is to create individual abilities and conditions of autonomy at the island level. The additional benefit is reduced travel and accommodation costs for patients and accompanying relatives. Indeed, between 10% and 15% of admissions to ICUs in Martinique and Guadeloupe are foreign patients, sometimes without health insurance. For this, cooperation with neighboring countries with local capacity-building is essential and could be cost-saving (22, 23). It is expected that this cooperation, together with the launch of a road map, will catalyze initiatives at regional, interregional, and national levels, with the support of PAHO/World Health Organization offices, ministries of health, and other stakeholders.

**French Guiana.** French Guiana is located in the Amazon region, and patients living in the Brazilian and Suriname riverside areas regularly seek treatment in the hospitals of French Guiana and are then transferred to the ICU in Cayenne Hospital when necessary. In the field of critical care, the nearest ICU in Suriname is in Paramaribo (336 km from Cayenne), and in Brazil it is in Macapa (Amapa state, 782 km from Cayenne). However, there is no formalized cooperation established between French Guiana and neighboring countries. Recently, cooperation with health authorities in Brazil has allowed experience exchange and patient management, mainly in the field of animal envenoming (12, 24).

## PROSPECTS

Looking forward, the promotion of intensive care in the FTA needs enhancement of the ICUs in the three sites with an upgrade of the ICU infrastructure in French Guiana and Guadeloupe. Indeed, the ICU admission capacity in the FTA is lower than in mainland France and Europe (3). This insufficient capacity became more evident during the COVID-19 crisis.

Also, the setting up of a complete technical platform is necessary for full autonomy of management of severe cases, to reduce the number of medevacs to mainland France, which are risky for unstable patients and costly. Recently, telemedicine systems have opened the door to closer working between the three teams and intensive care specialists in mainland France and in the Caribbean region. It has allowed experience exchange and e-learning, which have contributed to the improvement of practices and strengthened the collaboration between the three sites. In addition, the Caribbean doctors’ network was set up in 2020, with regular videoconferencing meetings to exchange experiences about COVID-19.

Finally, improving ICU capacity in the FTA requires the promotion of international medical and scientific cooperation with the neighboring countries in the Caribbean and in the Amazon region (25). For this, the Antilles-Guyane Association of Intensive Care Medicine was created in 2017.

## CONCLUSION

The presented data provide insights into the specificities and the distribution of intensive care resources in the FTA. We conclude that there is an urgent need to provide the FTA with more ICU beds to deal with recurrent epidemics, emerging and neglected tropical diseases, and environmental problems that are affecting the region. In addition, there is a need to formalize national and international cooperation with neighboring countries to promote critical care.

## Disclaimer.

Authors hold sole responsibility for the views expressed in the manuscript, which may not necessarily reflect the opinion or policy of the *RPSP/PAJPH* and/or PAHO.
